# *Arabidopsis thaliana* phytochelatin synthase 2 is constitutively active *in vivo* and can rescue the growth defect of the *PCS1*-deficient *cad1-3* mutant on Cd-contaminated soil

**DOI:** 10.1093/jxb/eru195

**Published:** 2014-05-12

**Authors:** Tanja Kühnlenz, Holger Schmidt, Shimpei Uraguchi, Stephan Clemens

**Affiliations:** Department of Plant Physiology, University of Bayreuth, Universitätsstrasse 30, 95447 Bayreuth, Germany

**Keywords:** *Arabidopsis*, metal tolerance, Cd tolerance, Cd contamination, PCS overexpression, phytochelatins, LC-MS.

## Abstract

Phytochelatins play a key role in the detoxification of metals in plants and many other eukaryotes. Their formation is catalysed by phytochelatin synthases (PCS) in the presence of metal excess. It appears to be common among higher plants to possess two *PCS* genes, even though in *Arabidopsis thaliana* only *AtPCS1* has been demonstrated to confer metal tolerance. Employing a highly sensitive quantification method based on ultraperformance electrospray ionization quadrupole time-of-flight mass spectrometry, we detected AtPCS2-dependent phytochelatin formation. Overexpression of AtPCS2 resulted in constitutive phytochelatin accumulation, i.e. in the absence of metal excess, both *in planta* and in a heterologous system. This indicates distinct enzymatic differences between AtPCS1 and AtPCS2. Furthermore, AtPCS2 was able to partially rescue the Cd hypersensitivity of the AtPCS1-deficient *cad1-3* mutant in a liquid seedling assay, and, more importantly, when plants were grown on soil spiked with Cd to a level that is close to what can be found in agricultural soils. No rescue was found in vertical-plate assays, the most commonly used method to assess metal tolerance. Constitutive AtPCS2-dependent phytochelatin synthesis suggests a physiological role of AtPCS2 other than metal detoxification. The differences observed between wild-type plants and *cad1-3* on Cd soil demonstrated: (i) the essentiality of phytochelatin synthesis for tolerating levels of Cd contamination that can naturally be encountered by plants outside of metal-rich habitats, and (ii) a contribution to Cd accumulation under these conditions.

## Summary

Experiments on Cd-contaminated soil demonstrated a contribution of phytochelatin synthesis to agriculturally relevant Cd accumulation and revealed constitutive activity of the hitherto functionally not understood *Arabidopsis thaliana* phytochelatin synthase2.

## Introduction

Due to their sessile nature, plants have to cope with various biotic and abiotic stress conditions. Changing availabilities of heavy metals are among the latter. They pose a threefold challenge for plants. First, essential micronutrients such as Fe, Zn, Cu, Mn, and Mo have to be acquired in sufficient amounts. Secondly, their toxic effects have to be limited when present in supraoptimal concentrations. Thirdly, the co-occurrence of non-essential heavy metals or metalloids such as Cd and As, which are simply toxic and taken up as a result of their chemical similarity to essential nutrients, i.e. through transport proteins with a limited substrate specificity, necessitates an array of rapid and flexible but also specific detoxification mechanisms.

One key component of the response to at least challenges two and three in all higher plants is the metal-activated synthesis of phytochelatins (PCs). These are small cysteine-rich peptides of the general structure (γ-Glu-Cys)_*n*_-Gly with chain lengths from *n*=2–7 (usually referred to as PC2–PC7) *in vivo* (Grill *et al.*, 1985). Their synthesis is catalysed by the enzyme phytochelatin synthase (PCS) in the presence of a range of heavy-metal ions, which are then complexed by the free thiol groups of the formed PCs (Grill *et al*., 1987, 1989). In *Arabidopsis thaliana*, this metal-dependent transpeptidation reaction is carried out by AtPCS1, encoded by the *CAD1* locus (Ha *et al.*, 1999). Loss of function of this gene results in a highly Cd-hypersensitive mutant, namely *cad1-3*. Later, it was found that *cad1-3* is also Zn hypersensitive (Tennstedt *et al.*, 2009). The *cad1-3* mutant was characterized as PC deficient (Howden *et al.*, 1995*b*). Therefore, it was unexpected when a second *PCS* gene, *AtPCS2*, was found (Cazalé and Clemens, 2001).

AtPCS2 shares 84% identity at the amino acid level with AtPCS1 and the exon–intron structure of the respective genes is similar. However, the intron sequences differ by 60–80%, suggesting a longer coexistence of both genes. The *AtPCS2* transcript can be detected in shoots and roots, albeit at much lower levels than for *AtPCS1* (Ha *et al.*, 1999; Cazalé and Clemens, 2001). Fusion proteins of AtPCS1 and enhanced green fluorescent protein (AtPCS1:eGFP) expressed under control of the endogenous promoter have been detected in the cytoplasm throughout the whole plant, while the respective AtPCS2:eGFP fusion showed a signal only in the root tip (Blum *et al.*, 2010).

To date, no function has been ascribed to AtPCS2 *in planta*. While heterologous expression of *AtPCS2* conferred Cd tolerance to *Schizosaccharomyces pombe* as well as *Saccharomyces cerevisiae* (Cazalé and Clemens, 2001) and thereby indicated that this gene does encode a functional PCS, overexpression in *cad1-3* led to at most a partial complementation upon Cd treatment (Lee and Kang, 2005). Moreover, the presence of a functional *PCS2* gene in *cad1-3* is not sufficient to confer Cd and Zn tolerance, as demonstrated by the pronounced hypersensitivity of this mutant. Finally, no mutant phenotype has yet been reported for an *atpcs2* mutant (Blum *et al.*, 2007).

More recently, *AtPCS1* has been implicated not only in Zn tolerance (Tennstedt *et al.*, 2009) but also in pathogen defence. A typical *A. thaliana* innate immune response—callose deposition activated by exposure to the microbe-associated molecular pattern flagellin (flg22)—was reported to be dependent on *AtPCS1* (Clay *et al.*, 2009). Both functions suggest PCS activities in the absence of toxic metal stress and a physiological role also of constitutive low-level PC synthesis. Therefore, the ability to detect PCs with high sensitivity is necessary. Furthermore, it is important to elucidate the possible contribution of AtPCS2 to PC synthesis in *A. thaliana.*

In this study, a highly sensitive method for PC2 and PC3 detection using ultraperformance electrospray ionization quadrupole time-of-flight mass spectrometry (UPLC-ESI-QTOF-MS) is reported. This analytical approach enabled the detection of background levels of PC2 in *cad1-3*, which were absent in the generated *cad1-3 atpcs2* double mutant. This strongly suggests AtPCS2-dependent constitutive PC synthesis. Furthermore, it was found that AtPCS2 was able to rescue the Cd hypersensitivity of *cad1-3* when tested on Cd-contaminated soil. Interestingly, AtPCS2-dependent PC synthesis was not enhanced by the presence of Cd^2+^, which is in contrast to what was observed for AtPCS1.

## Materials and methods

### Plant lines

An *A. thaliana* T-DNA line with an insertion in an exon of the *AtPCS2* gene was available from public repositories in the Wassilewskija (Ws-0) background only (Alonso *et al.*, 2003; http://signal.salk.edu/cgi-bin/tdnaexpress, last accessed 22 April 2014). Homozygous *atpcs2* lines (FLAG_146G12) (Samson *et al.*, 2002) were identified via PCR using the T-DNA border primer TAG5 (5′-CTACAAATTGCCTTTTCTTATCGAC-3′) and the gene-specific primer PCS2_rev (5′-GCAGATTGT CTTCGTACACAGAGG-3′). For detection of the wild-type fragment, PCS2_rev was combined with primer PCS2_fw (5′-GATGAATCAATGCTGGAATGTTGC-3′) (Supplementary Fig. S1 at *JXB* online). The *PCS* single mutants *cad1-3* (Howden *et al.*, 1995*b*) and *atpcs2* were crossed to obtain the double mutant *cad1-3 atpcs2*. Homozygous lines were identified with the above-mentioned primer combinations for the *AtPCS2* locus. The G/C nucleotide exchange in the *cad1-3* mutant was scored by PCR with the primer pair AtPCS1_fw (5′-CCGCAAATTTGTCGTCAAATG-3′) and cad1-3_mut._rev (5′-CCCAAAGAAGTTTAAGAGGACCG-3′) (annealing at 62 °C), which contained in addition to the mutated C nucleotide at the 3′ end a second mismatched base at position 3 from the 3′ end. The wild-type allele was identified using the primer combination cad1-3_wt_fw (5′-CAAGTATCCCCCTCACCGG-3′) and AtPCS1_rev (5′-CATGAACCTGAGAACAACACAGA-3′) (annealing at 61 °C). For a quality check of the genomic DNA, the primer pair AtPCS1_fw/AtPCS1_rev was used.

The genetic backgrounds of *cad1-3* and *atpcs2* are Col-0 and Ws-0, respectively. Col-0 and Ws-0 are known to differ in a gene related to Cd transport. Col-0 carries a single base-pair deletion in *AtHMA3*, a P_1B_-ATPase gene that influences shoot Cd accumulation (Chao *et al.*, 2012). As the *cad1-3 atpcs2* double mutant was obtained by crossing of the respective single mutants in the Col-0 or Ws-0 background, the genotype of *AtHMA3* in the double mutant was verified by a cleaved amplified polymorphic sequence (CAPS) marker based on the single-nucleotide deletion in the Col-0 allele. An *AtHMA3* fragment was amplified by PCR using the primer pair AtHMA3_CAPS_fw (5′-AGAGAGCTGGATGCTTAACAGGTC-3′) and AtHMA3_CAPS_rev (5′-TACCATCATTGTTGGCCCTTG-3′), followed by *Dde*I (NEB) digestion. The double mutant used in this study was homozygous for the Col-0 allele of *AtHMA3* (Supplementary Fig. S2 at *JXB* online).

Generation of lines overexpressing *AtPCS1*, driven by the cauliflower mosaic virus 35S promoter in the *cad1-3* background, has been described previously (Tennstedt *et al.*, 2009). *AtPCS2*-overexpression lines were obtained correspondingly. Both gene constructs add a haemagglutinin (HA) tag C-terminally (*AtPCS1*-HA and *AtPCS2*-HA) and were tested for functionality previously by expression in *Schizosaccharomyces pombe* (Cazalé and Clemens, 2001).

### Plant growth

Seeds of *A. thaliana* were surface sterilized by exposure to chlorine gas (produced by adding 5ml of 32% HCl to 10ml of sodium hypochlorite solution) in an desiccator for 35min.

The plant growth medium used was based on one-tenth-strength modified Hoagland’s solution No. 2 [0.28mM Ca(NO_3_)_2_, 0.6mM KNO_3_, 0.1mM NH_4_H_2_PO_4_, 0.2mM MgSO_4_, 4.63 µM H_3_BO_3_, 32nM CuSO_4_, 915nM MnCl_2_, 77nM ZnSO_4_, 11nM MoO_3_] (Hoagland and Arnon, 1950). Fe was supplied as *N,N′*-di-(2-hydroxybenzoyl)-ethylenediamine-*N,N′*-diacetic acid according to the method of Chaney (1988) to a final concentration of 5 µM. Tolerance assays were performed without microelements, except for iron, either in a liquid seedling assay or on agar plates in medium containing 1% (w/v) sucrose, 0.05% (w/v) MES at pH 5.7. Cd^2+^ was added as the chloride salt. Liquid seedling assays were performed in six-well tissue culture plates with six seeds per well in 5ml of medium. For agar plate assays, the medium was solidified with 1% (w/v) Agar Type A (Sigma-Aldrich). Plates of both assay types were sealed with Leucopore tape (Duchefa). Following stratification for 2 d at 4 °C, the seedlings were incubated under long-day conditions (16h light/8h dark) at 22 °C. The agar plates were placed vertically and the liquid assay was shaken gently.

For PC analysis, plants were grown hydroponically for 6.5 weeks under short-day conditions (8h light, 22 °C/16h dark, 18 °C) in one-tenth-strength modified Hoagland’s solution including all microelements without sucrose and modified concentrations of Ca(NO_3_)_2_ (0.4mM) and (NH_4_)_2_HPO_4_ (0.0871mM). Cultivation started in agar-filled PCR tubes in pipette tip boxes for 3 weeks followed by transfer into 50ml tubes (Greiner Bio-One) for another 3 weeks before addition of 0.5 µM or 5 µM CdCl_2_. No additional heavy-metal ions were added to the respective controls. To guarantee a sufficient oxygen and mineral supply, the medium was changed weekly throughout cultivation.

Experiments on artificially contaminated soil were conducted under short-day conditions (8h light, 22 °C/16h dark, 18 °C) using a mineral soil type that was prepared as follows. After drying the soil at 70 °C, organic compounds were removed to the greatest possible extent by sieving. Five hundred grams of soil (dry weight) in plastic bottles and 250ml of water containing 10mg CdCl_2_ monohydrate were mixed thoroughly for 1h in an overhead shaker (Reax 20/12; Heidolph) until a homogeneous soil solution was obtained. Control soil was mixed with water only. Afterwards, the soil was dried, sieved again, and mixed to one-third with sand featuring a low ion-binding capacity. In order to achieve a high level of comparability, pots were filled with equal amounts of the prepared soil and moistened with the same volume of water. After loosening the humid soil, seedlings were transferred into this soil 10 d after germination on solid one-tenth-strength Hoagland medium. Plants were grown in a randomized manner for 24 d and all plants were irrigated daily with equal volumes of water. Furthermore, in order to minimize desiccation gradients that could influence metal availability and uptake, experimental plants were surrounded by a belt of control plants that were excluded from data acquisition. At the end of the experiment, two sample pools per plant line and condition were formed in the sense that equivalent phenotypic variance was obtained between both pools. After removal of soil particles by washing with Millipore water, the pooled leaf material was frozen in liquid nitrogen.

### Quantification of leaf area

The growth of plants on artificially contaminated soil was tracked by quantification of the leaf area. Single plants were photographed every 7 d after the transfer to soil and at the end of the experiment. Leaf area was measured using Adobe Photoshop CS2 version 9.0 by counting the number of pixels representing the leaf shape and normalizing to the number of pixels per pot.

### Elemental analysis via inductively coupled plasma optical emission spectroscopy

One part of the pooled leaf material from soil-grown plants was freeze dried and digested in 4ml of 65% HNO_3_ and 2ml of 30% H_2_O_2_ using a microwave (START 1500; MLS GmbH). Cd content was measured with an iCAP 6500 (Thermo Scientific) at a wavelength 226.5nm. For the determination of extractable and exchangeable soil metal contents, 3g of dried and sieved soil was extracted with 25ml of the respective solution using a rotator (SB2, Stuart). The extractable metal content was analysed in extracts with 0.1M HCl (30min, 23 °C) (Kuo *et al.*, 2006), whereas the exchangeable portion was measured after incubation for 2h at 23 °C with either 10mM CaCl_2_ (Houba *et al.*, 2000) or 5mM diethylenetriaminepentaacetic acid (DTPA) containing 0.1M triethanolamine and 10mM CaCl_2_ (pH 7.3) (Lindsay and Norvell, 1978). If required, samples were diluted in 2% HNO_3_ for the metal analysis.

### PC analysis

PC concentrations were measured in plant material obtained from three different experimental setups: (i) whole seedlings after 11 d of growth in liquid one-tenth-strength Hoagland medium, control or exposed to 0.5 µM CdCl_2_; (ii) leaf and root material from 6.5-week-old plants grown in hydroponic culture, untreated or treated for 3 d with 0.5 µM or 5 µM CdCl_2_; and (iii) leaf material after 24 d of growth on soil with or without 7.5mg Cd^2+^ kg^–1^ of soil. All plant material was frozen in liquid nitrogen and ground to a homogenous powder. One hundred milligrams (fresh weight) was extracted with 300 µl of 0.1% (v/v) trifluoroacetic acid containing 6.3mM DTPA (Sneller *et al.*, 2000) and 40 µM *N*-acetylcysteine (NAC) as an internal standard. The plant material was suspended completely by exhaustive mixing. The homogenate was chilled on ice for 15min and mixed occasionally. Following centrifugation (16 000*g*, 15min, 4 °C), the PC-containing supernatant was reduced and derivatized based on the methods of Rijstenbil and Wijnholds (1996) and Sneller *et al.* (2000), as described by Thangavel *et al.* (2007) and Minocha *et al.* (2008). Derivatization was performed to achieve a better retention on reversed-phase column material. Briefly, 62.5 µl of the extract or a mix of the respective standards was added to 154 µl 200mM 4-(2-hydroxyethyl)-piperazine-1-propanesulfonic acid (EPPS) (6.3mM DTPA, pH 8.2) and 6.25 µl 20mM Tris-(2-carboxyethyl)-phosphine (TCEP; prepared freshly in 200mM EPPS, pH 8.2) and incubated for 10min at 45 °C. Afterwards, 5 µl of 50mM monobromobimane (mBrB; in acetonitrile) started the labelling reaction, which was performed for 30min at 45 °C. The reaction was stopped by the addition of 25 µl of 1M methanesulfonic acid. In contrast to former methods (Rijstenbil and Wijnholds, 1996; Sneller *et al.*, 2000; Thangavel *et al.*, 2007; Minocha *et al.*, 2008), the internal standard NAC was already present in the extraction buffer in order to minimize variation between samples. Samples were centrifuged at 16 000*g* at 4 °C prior to analysis. A Waters Aquity UPLC system equipped with an HSS T3 column (1.8 µm, 2.1×100mm; Waters Corporation, Milford, MA) was used for the separation of the mBrB-labelled thiols. The injection volume was 5 µl. A 15min linear binary gradient of water (A) and acetonitrile (B), both acidified with 0.1% (v/v) formic acid, at a flow of 0.5ml min^–1^ was employed: 99.5% A, 0.5% B for 1min, a linear gradient to 60.5% B at 10min, gradient to 99.5% B at 12min, flushing with 99.5% B for 1min, a gradient back to initial conditions in 1min and an additional re-equilibration for 1min. The column temperature was set to 40 °C. Thiols were detected with a Q-TOF Premier mass spectrometer equipped with an ESI-source (Waters Corporation) operated in the V+ mode. The mass spectrometer was tuned for optimal sensitivity using leucine enkephalin. Basic parameters were: capillary 0.6kV, sampling cone 30V, extraction cone 30V, ion guide 3.3V, source temperature 120 °C, cone gas flow 10 l h^–1^, desolvation gas flow 1000 l h^–1^, collision energy 4.0V. Data were acquired from *m*/*z* 300–2000 with a scan time of 0.3 s and an inter-scan delay of 0.05 s. For quantification the QuanLynx module of the MarkerLynx software was used. PC2 and PC3 were quantified by integration of the reconstructed ion traces of the protonated ions [M+H]^+^ *m*/*z* 354.1±0.5 at 4.39±0.3min for mBrB–NAC (internal standard), *m*/*z* 920.3±0.5 at 4.57±0.3min for mBrB–PC2 and the added ion traces of *m*/*z* 1342.4 + [M+2H]^2+^, and *m*/*z* 671.7±0.5 at 5.06±0.3min for mBrB–PC3. Using a linear calibration forced through the origin (125, 250, and 500nM and 2.5, 5, and 12.5 µM PC), the average *R* ^2^ was 0.98 for PC2 and 0.96 for PC3. All samples were measured in three technical replicates.

### Western blotting

For detection of HA-tagged PCS proteins, leaf material was extracted with 50mM sodium phosphate buffer (pH 8.0) containing 0.5mM ditiothreitol, 0.1× protease inhibitor mix (Roche), 0.1% (w/v) SDS and 0.1% (v/v) Triton-X-100. Total protein contents were determined by the bicinchoninic acid method (ThermoScientific). Protein extracts were separated on a denaturing 12% polyacrylamide gel and transferred onto a nitrocellulose membrane (Protran^TM^; Whatman). The PCS proteins were detected with monoclonal anti-HA antibody (Sigma-Aldrich; 1:3000 diluted) and stained using anti-mouse antibody (Sigma-Aldrich, 1:10 000 diluted) coupled to horseradish peroxidase and using the enhanced chemiluminescence method.

### Transcript analysis

Total RNA extraction from whole seedlings and quantitative real-time RT-PCR were performed as described previously (Deinlein *et al.*, 2012). cDNA synthesis was performed using a first-strand cDNA synthesis kit (Thermo Scientific) followed by quantitative real-time PCR using iQ SYBR Green supermix (BioRad). In order to evaluate the Zn and Cu status of seedlings grown in the modified Hoagland solution without microelements other than Fe, *ZIP9* (At4g33020), and *CCH* (At3g56240) and *COX5b-1* (At3g15640) were used as established molecular markers for Zn and Cu deficiency, respectively (Talke *et al.*, 2006; Bernal *et al.*, 2012). *Elongation factor 1α* (At5g60390) served as a reference gene. For primer sequences, see Supplementary Table S2 at *JXB* online.

### Heterologous expression of *AtPCS1* and *AtPCS2* in *Schizosaccharomyces pombe*

The *Schizosaccharomyces pombe PCS* knockout strain Δ*pcs* carrying the vector constructs pSGP72-*AtPCS1-HA* or pSGP72-*AtPCS2-HA* were used for heterologous expression of PCS proteins (Clemens *et al.*, 1999; Cazalé and Clemens, 2001). Cells carrying the empty vector served as negative control. Yeast cultivation was carried out at 30 °C in Edinburgh’s minimal medium (EMM; Moreno *et al.*, 1991) containing 20 µM thiamine to suppress *PCS* expression. To monitor thiol profiles, pre-cultured cells were inoculated to an optical density at 600nm (OD_600_) of 0.05 in EMM supplemented with 20 µM thiamine and grown overnight. The cells were then washed twice in thiamine-free EMM and inoculated at an OD_600_ of 0.4 in thiamine-free EMM in the presence or absence of extra metal ions. After 6h incubation, the cells were harvested and lyophilized. Extraction, labelling and quantification of PCs were carried out as described above for plant samples.

For detection of the HA-tagged PCS proteins, protein extracts were prepared from cells before and after 6h cultivation in thiamine-free EMM in the absence of Cd^2+^. Western blot analysis was performed as described above.

### Statistical analysis

Statistical analyses were performed with SigmaPlot 11.0.

## Results

### Analysis of PCS2 presence in the plant kingdom

**Fig. 1.**
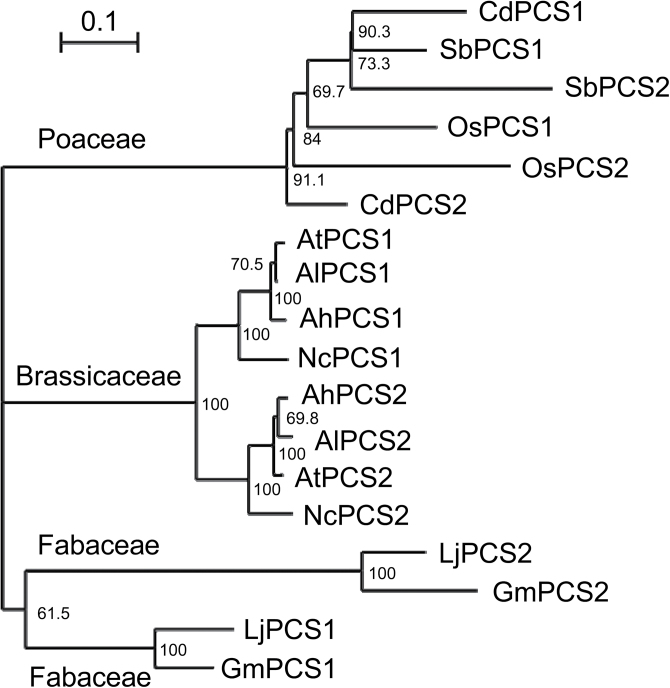
A second PCS is encoded in the genome of monocot and dicot species. PCS1 and PCS2 protein sequences available for species representing the Poaceae, Brassicaceae, and Fabaceae were subjected to hierarchical cluster analysis. The dendrogram was generated on the basis of a ClustalW+ protein sequence alignment using the protein maximum-likelihood (PhyML) algorithm (http://www.trex.uqam.ca, last accessed 22 April 2014; Boc *et al.*, 2012). Sequences included in the analysis are listed as follows (GenBank accession numbers in brackets): *Cynodon dactylon* (AAO13810, AAS48642), *Sorghum bicolor* (XP_002454970, XP_002454971), *Oryza sativa* (EEE64936, NP_001055554), *Arabidopsis lyrata* (XP_002865384, XP_002892190), *Arabidopsis halleri* (AAS45236, ADZ24787), *Arabidopsis thaliana* (NP_199220, AAK94671), *Noccaea caerulescens* (AAT07467, ABY89660), *Lotus japonicus* (AAT80342, AAT80341),and *Glycine max* (NP_001235576, XP_003537353). Bar, 0.1 is equal to 10% sequence divergence. Numbers indicate bootstrap values (%).

Few studies to date have reported evidence for the existence of a second *PCS* gene in species other than *A. thaliana*. Legumes such as *Lotus japonicus* were found to carry three *PCS* genes probably originating from two duplication events (Ramos *et al.*, 2007). Metal-hyperaccumulating Brassicaceae *Arabidopsis halleri* and *Noccaea caerulescens* both possess two *PCS* genes like their relative *A. thaliana* (Meyer *et al.*, 2011). Therefore, a search for *PCS2*-like genes among sequenced plant genomes was performed. Analysis of derived amino acid sequences revealed separate clusters of PCS1-like and PCS2-like proteins in Brassicaceae and Fabaceae (Fig. 1). Two distinct *PCS* genes were also found in monocot genomes. Thus, the presence of at least two *PCS* genes appears to be a general feature of plant genomes. This reinforces the need to functionally understand the role of *PCS2* genes.

### Development of a sensitive method for PC detection

**Fig. 2.**
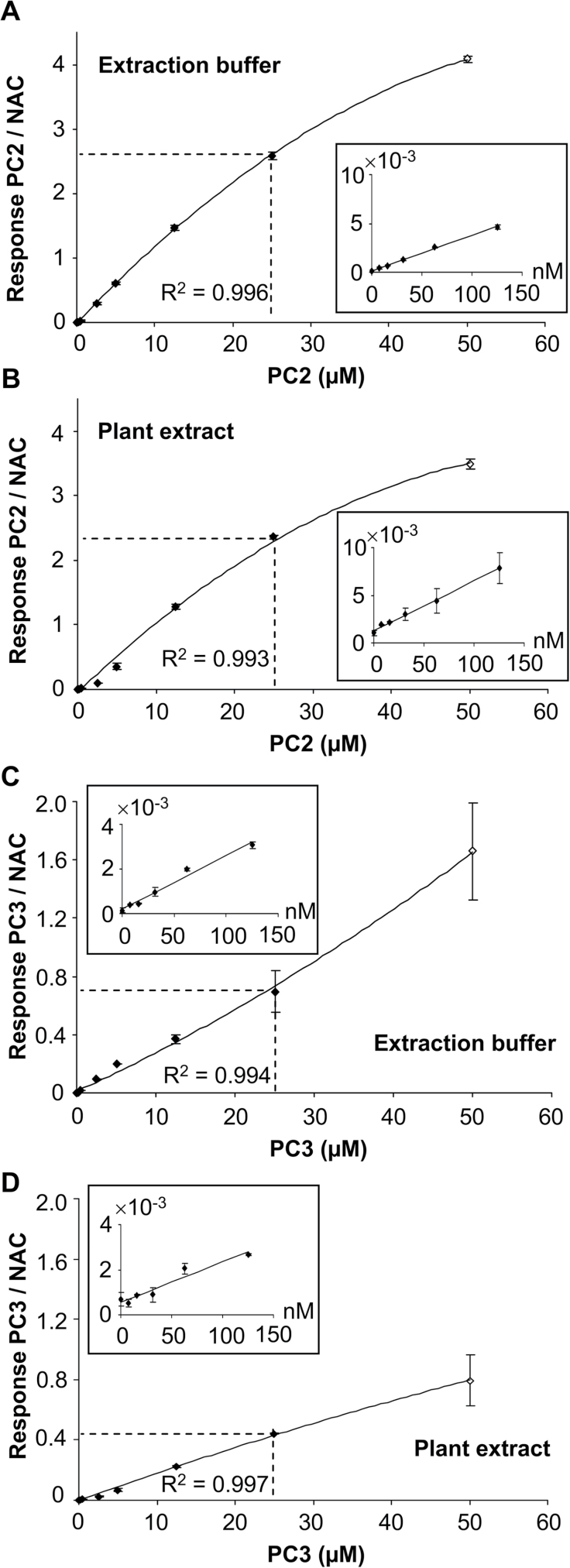
Linear range, recovery, and limits of detection for PC analysis via UPLC-ESI-QTOF-MS in plant matrix. Mixtures of PC2–PC5 standards were added either to extraction buffer [6.3mM DTPA+0.1% (v/v) trifluoroacetic acid] or to leaf extracts from Col-0 plants grown hydroponically under control conditions. Samples were reduced with TCEP prior to mBrB derivatization of thiol groups. Shown is the TOF-MS response for PC2 (A, B) and PC3 (C, D) relative to the internal standard NAC at the adjusted final PC concentrations in extraction buffer (A, C) or leaf extract (B, D). The correlation coefficient is indicated for the linear detection range up to 25 µM PC2 or PC3. The inset shows a plot of the PC:NAC ratio in the nanomolar range. Data represent means±standard deviation (SD) of three technical replicates. Lower limits of detection (signal:noise ratio > 3) of 50 and 100fmol for PC2 and PC3, respectively, were determined.

The contribution of AtPCS2 to metal homeostasis and tolerance is to date unclear. No evidence for *in planta* transpeptidase activity, which should be discernible as PC production in *cad1-3*, has been reported yet. In order to evaluate if and how AtPCS2 contributes to the PC pool and thereby potentially to the buffering of free metal ions within the cell, it was necessary to establish a highly sensitive method for the detection of trace amounts of PCs. Coupling of reversed-phase LC to ESI-QTOF-MS enabled such PC detection (Bräutigam *et al.*, 2009; Simmons *et al.*, 2009; Tennstedt *et al.*, 2009). In this study, mBrB-derivatized thiols, reduced with TCEP, were analysed via UPLC-ESI-QTOF-MS. The detection and quantification limits of the system were determined by examining the recovery rates of PC2–PC5 over a broad concentration range from 7.8nM to 50 µM in extraction buffer and in matrix (Col-0 leaf extracts). Fig. 2 shows the correlations between the adjusted final concentrations of PC2 and PC3 injected, and the detector response relative to the internal standard NAC. Broken lines indicate the linear ranges with an *R* ^2^>0.99. They extended well into the nanomolar range (see insets in Fig. 2) and were not influenced by leaf extracts. Matrix effects were apparent, however, when comparing recovery rates, i.e. slopes of the response curves in the linear range. Leaf matrix slightly reduced the PC2 response (by about 9%) and had a more pronounced effect on PC3 (reduction of about 37%). The saturation that the response curves for PC2 and PC3 showed at concentrations >25 µM was negligible because concentrations above 13 µM were never observed, even in extracts derived from Cd^2+^-treated plants.

Lower limits of detection (LLOD; signal:noise ratio >3) of 50 and 100fmol for PC2 and PC3, respectively, were determined. This translates into about a 4000-fold gain in sensitivity compared with the LLOD for PC2 obtained by conventional HPLC analysis as reported by Howden *et al.* (1995*b*) and applied for the initial characterization of the *cad1-3* mutant. Compared with studies employing HPLC of mBrB-derivatized PCs or LC-MS analyses of non-derivatized PCs, a gain of between about 20-fold (Simmons *et al.*, 2009) and about 2-fold (Minocha *et al.*, 2008) was achieved. PC4 and PC5, which were part of the standard mix, were not included in later PC quantification due to the high *m*/*z* ratio of their protonated ions of [M+H]^+^ 1764.5 and 2186.7, respectively. The necessary expansion of the routinely used calibration range (*m*/*z* 300–2000) and scan time would have resulted in a loss of sensitivity for PC2 and PC3. Moreover, the summed ion traces of [M+H]^+^ and [M+2H]^2+^ for PC4 and PC5 constituted maximally a small proportion of the total PC concentrations found.

### Detection of PCs in *cad1-3*

Equipped with this highly sensitive method for PC detection, the contribution of AtPCS2 to PC accumulation was re-examined. A T-DNA line in the Ws-0 background with an insertion in exon 6 of *AtPCS2* was obtained (Supplementary Fig. S1). Also, a double mutant was generated by crossing this *atpcs2* mutant line with *cad1-3*. The genetic backgrounds of *cad1-3* and *atpcs2* are Col-0 and Ws-0, respectively. Because Col-0 in contrast to Ws-0 does not carry a functional allele of *AtHMA3*, a P_1B_-ATPase gene that influences shoot Cd accumulation (Chao *et al.*, 2012), we selected a double mutant that was homozygous for the Col-0 allele of *AtHMA3* (Supplementary Fig. S2).

**Fig. 3.**
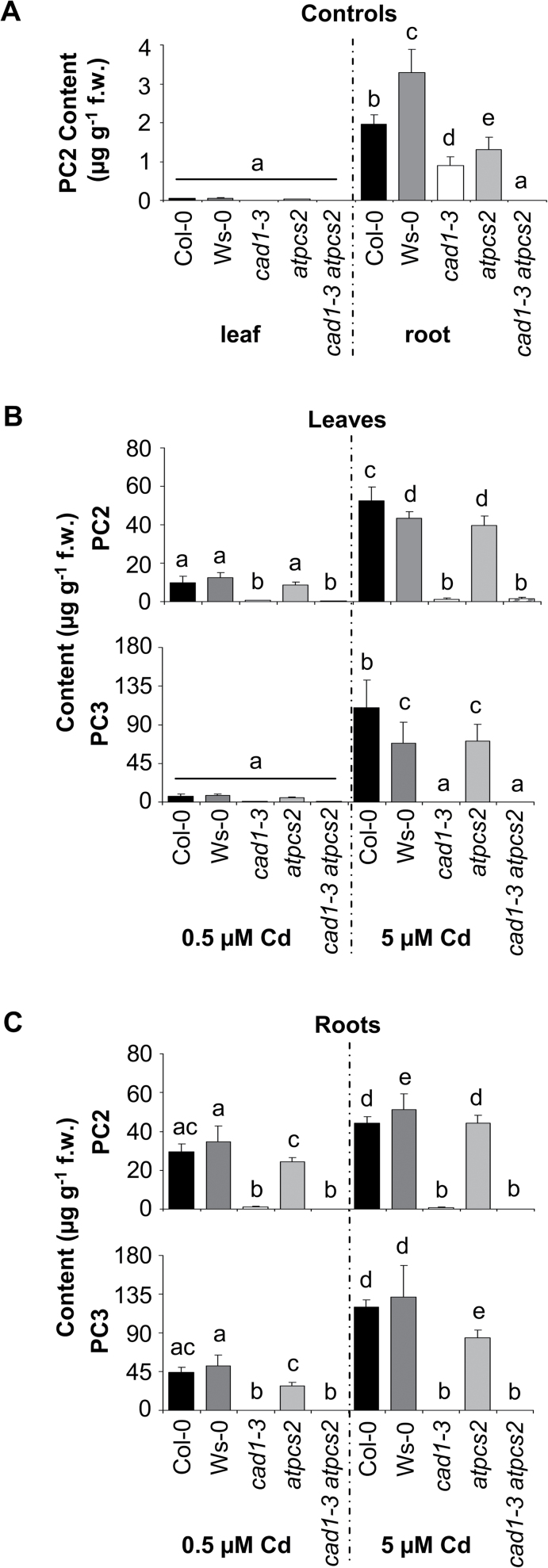
Detection of PC2 formation in *cad1-3*. The *A. thaliana AtPCS1* and *AtPCS2* single mutants *cad1-3* and *atpcs2* as well as their respective wild types Col-0 and Ws-0 and the double mutant *cad1-3 atpcs2* were grown hydroponically in one-tenth-strength Hoagland medium with all micronutrients for 6.5 weeks. PC accumulation was analysed by UPLC-ESI-QTOF-MS. (A) PC2 concentrations for plants grown in control medium. Note that, except for PC2 in roots, all values measured under control conditions were below the lower limit of quantification. (B, C) Plants were exposed to 0.5 or 5 µM CdCl_2_ for 3 d. Leaves (B) and roots (C) were analysed separately. Data represent means±SD of two independent experiments with six samples in total per plant line and condition. For each sample, material from three plants was pooled. Data were statistically analysed via two-way analysis of variance (ANOVA) and grouped with Tukey’s 95% confidence interval. f.w., Fresh weight.

Single mutants, the double mutant, and the respective wild types Col-0 and Ws-0 were grown in hydroponic culture and treated for 3 d with 0.5 or 5 µM CdCl_2_ in addition to the regular microelement concentrations. Leaf and root material was analysed separately for PC accumulation via UPLC-ESI-QTOF-MS (Fig. 3). In roots of plants cultivated under control conditions, PC2 was detectable in both wild types and the single mutants, thus demonstrating PC formation even in the absence of any metal excess (Fig. 3A). PC3 was not detected in root extracts. No PCs above the lower limit of quantification (LLOQ; signal:noise ratio > 10) were found in leaves of plants grown in the absence of Cd^2+^. As expected, Cd^2+^-exposed wild-type plants showed a strong increase in PC accumulation. Following addition of 0.5 µM Cd^2+^, root PC levels were 3-fold (PC2) and 6-fold (PC3) higher than in leaves. At the external Cd^2+^ concentration of 5 µM, this difference disappeared and PC levels in the roots and shoots were similar.

Interestingly, while no growth defect of *atpcs2* relative to Ws-0 was detected (data not shown), significant differences in PC2 and PC3 accumulation between the mutant and its corresponding wild type were found. In the presence of an external Cd^2+^ concentration of 5 µM, *atpcs2* accumulated PC2 in roots to levels comparable to Col-0 but less than Ws-0. PC3 concentrations were lower than in both wild types. The same trend was visible at 0.5 µM Cd^2+^, which suggested a measurable contribution of AtPCS2 to PC accumulation. In leaves, no differences in PC accumulation between *atpcs2* and Ws-0 were apparent.

More direct evidence for AtPCS2-dependent PC synthesis in roots came from the observation that PC2 accumulated in the leaves and roots of Cd^2+^-treated *cad1-3* plants to levels above the LLOQ (signal:noise ratio >10) (Fig. 3A–C). In contrast, no PC2 was detectable in any of the root samples derived from the double mutant. Leaf PC2 concentrations in the double mutant were below the LLOQ for plants exposed to 0.5 µM Cd^2+^. In the presence of 5 µM Cd^2+^, low amounts of PC2 could be detected in leaves. These were reduced compared with those found in *cad1-3*. No PC3 was detectable in *cad1-3* and *cad1-3 atpcs2*. Only in leaves in the presence of 0.5 µM Cd^2+^ were trace amounts of PC3 found, but these were below the LLOQ.

### Comparison of different metal tolerance assays reveals distinct phenotypes

**Fig. 4.**
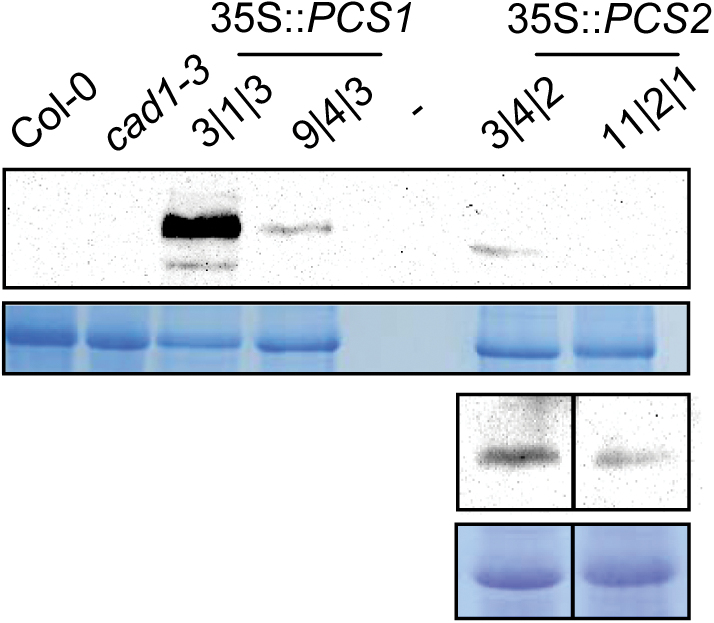
Transgenic *cad1-3* lines expressing either *AtPCS1* or *AtPCS2* under the control of the 35S promoter. Leaf protein extracts of the wild-type Col-0, the *AtPCS1* mutant *cad1-3* and 35S overexpression lines in the mutant background were analysed via SDS-PAGE, western blotting, and immunostaining. C-terminally HA-tagged versions of AtPCS1 (58kDa) and AtPCS2 (55kDa) were detected in the respective 35S lines with an anti-HA antibody. The Coomassie-stained SDS gel is shown as a loading control below the blot. No AtPCS2 signal was obtained for the weak line 11|2|1 when loaded next to the strong AtPCS1 line 3|1|3 (top). Only by loading on a separate gel (bottom) could a weak band be detected. For better comparison, lanes of lines 3|4|2 and 11|2|1, analysed on the same blot, were pasted side by side. (This figure is available in colour at *JXB* online.)

The evidence suggesting AtPCS2-dependent PC accumulation *in planta* prompted the investigation of a possible rescue of *cad1-3* through *AtPCS2* overexpression. Respective lines expressing *AtPCS2* under the control of the 35S promoter were generated and compared with *cad1-3* lines transformed with a corresponding construct carrying *AtPCS1*. Proteins were expressed with a C-terminal HA tag. Two lines each were selected that differed in protein expression as assessed by western blotting and immunostaining (Fig. 4). When analysed in vertical-plate assays, which clearly reveal the phenotypes of *cad1-3* seedlings (Tennstedt *et al.*, 2009), rescue of *cad1-3* was observed only in 35S::*AtPCS1* lines (Supplementary Fig. S3 at *JXB* online). For lines expressing *AtPCS2*, no more than a slight beneficial effect on shoot development was discernible.

It is now well documented that growth medium and conditions can strongly influence the emergence of metal-related phenotypes (Tennstedt *et al.*, 2009; Gruber *et al.*, 2013). Therefore, the *cad1-3* complementation was tested in additional assays. First, a newly established liquid seedling assay was used. In accordance with the medium conditions of the plate assays (Tennstedt *et al.*, 2009), seedlings were grown in one-tenth-strength Hoagland medium without micronutrients except for Fe. Quantitative real-time PCR tests with established markers for Zn (*ZIP9*; Talke *et al.*, 2006) and Cu deficiency (*CCH*, *COX5b-1*; Bernal *et al.*, 2012) showed that no micronutrient deficiency developed in Col-0 or *cad1-3* seedlings during the 7 d of cultivation in this assay. Relative transcript levels were only marginally different (Supplementary Table S3 at *JXB* online).

**Fig. 5.**
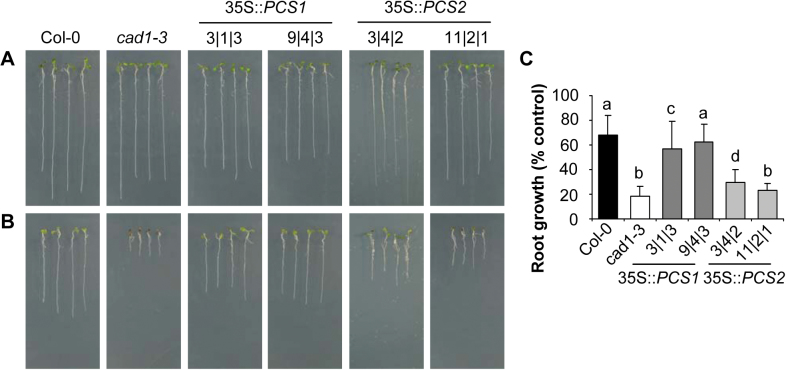
Rescue of *cad1-3* Cd hypersensitivity in liquid seedling assays by expression of *AtPCS1* or *AtPCS2.* (A–C) Seedlings of wild-type Col-0 (black bars), the *AtPCS1* mutant *cad1-3* (white bars) and lines overexpressing AtPCS1 (dark grey bars) or AtPCS2 (light grey bars) in the *cad1-3* background were germinated and grown in a liquid seedling assay in one-tenth-strength Hoagland medium either without metal addition (A) or in the presence of 0.5 µM CdCl_2_ (B). Seedlings were placed on agar plates before taking the pictures. Root length (C) was determined after 7 d growth under long-day conditions. Data represent means±SD of 4–11 independent experiments with 65–150 analysed individuals each in total. Percentage values were arc sine square root transformed prior to one-way ANOVA and grouped with Tukey’s 95% confidence interval. (This figure is available in colour at *JXB* online.)

Indeed, the change from solidified to liquid medium indicated partial rescue of *cad1-3* by *AtPCS2* overexpression (Fig. 5B). Due to differences between the lines under control conditions (Fig. 5A), it was necessary to compare their growth in the presence of Cd^2+^ as relative values, as the percentage of growth under control conditions (Fig. 5C). The primary root length of *cad1-3* following Cd^2+^ treatment reached 18% (±8%) of the root growth under control conditions, while roots of the 35S::*AtPCS2* lines 3|4|2 and 11|2|1 grew to 30 and 23%, respectively. The difference was significant for the 35S::*AtPCS2* line 3|4|2 (*P*<0.001). The second line, 11|2|1, with only weak *AtPCS2* expression, was not significantly different from *cad1-3* (*P*=0.106) but nevertheless tended to have longer roots than *cad1-3* in the presence of Cd^2+^. Mutant lines overexpressing *AtPCS1* showed a growth behaviour comparable to that of wild-type seedlings.

To test whether this partial complementation of *cad1-3* by 35S::*AtPCS2* overexpression was associated with a higher production of PCs, seedlings were analysed for their PC content after growth in the absence or presence of Cd^2+^ (Supplementary Fig. S4 at *JXB* online). *AtPCS1-*overexpressing lines 3|1|3 and 9|4|3 contained similar amounts of PC2 and PC3 and showed a slightly increased PC accumulation compared with the wild-type Col-0 following Cd^2+^ exposure. In addition, the 35S::*AtPCS2* lines 3|4|2 and 11|2|1 were found to produce PC2 and PC3, while in *cad1-3* only trace amounts of PC2 were detected. PC2 levels in 35S::*AtPCS2* 3|4|2 were comparable to Col-0, while the weakly expressing line 11|2|1 contained only 40% of the wild-type PC2 level. Both 35S::*AtPCS2* lines were strongly impaired in the production of the longer-chain PC3 relative to the wild-type and the 35S::*AtPCS1* lines. Interestingly, 35S::*AtPCS2* lines showed a 6-fold or 3-fold higher PC2 production in the absence of additional Cd^2+^ compared with Col-0. Constitutive PC2 production was also higher than in 35::*AtPCS1* seedlings.

Next, the growth behaviour of representative lines complementing *cad1-3* at least partially was tested under conditions that resembled a natural habitat far more closely than *in vitro* assays. For this, a mineral soil with a low organic content was artificially contaminated with Cd to a final concentration of 7.5mg kg^–1^ of soil. Extractable Cd as determined via the HCl method was 6.3±0.3mg Cd kg^–1^ of soil. The bioavailable Cd was estimated through extraction with DTPA or exchange with CaCl_2_ which yielded a value of 4.3±0.3mg and 0.12±0.02mg Cd kg^–1^ of soil, respectively. In control soil, only traces of Cd below the limit of quantification (0.01mg kg^–1^ of soil) were detectable. Concentrations of other metals are listed in Supplementary Table S1 at *JXB* online.

**Fig. 6.**
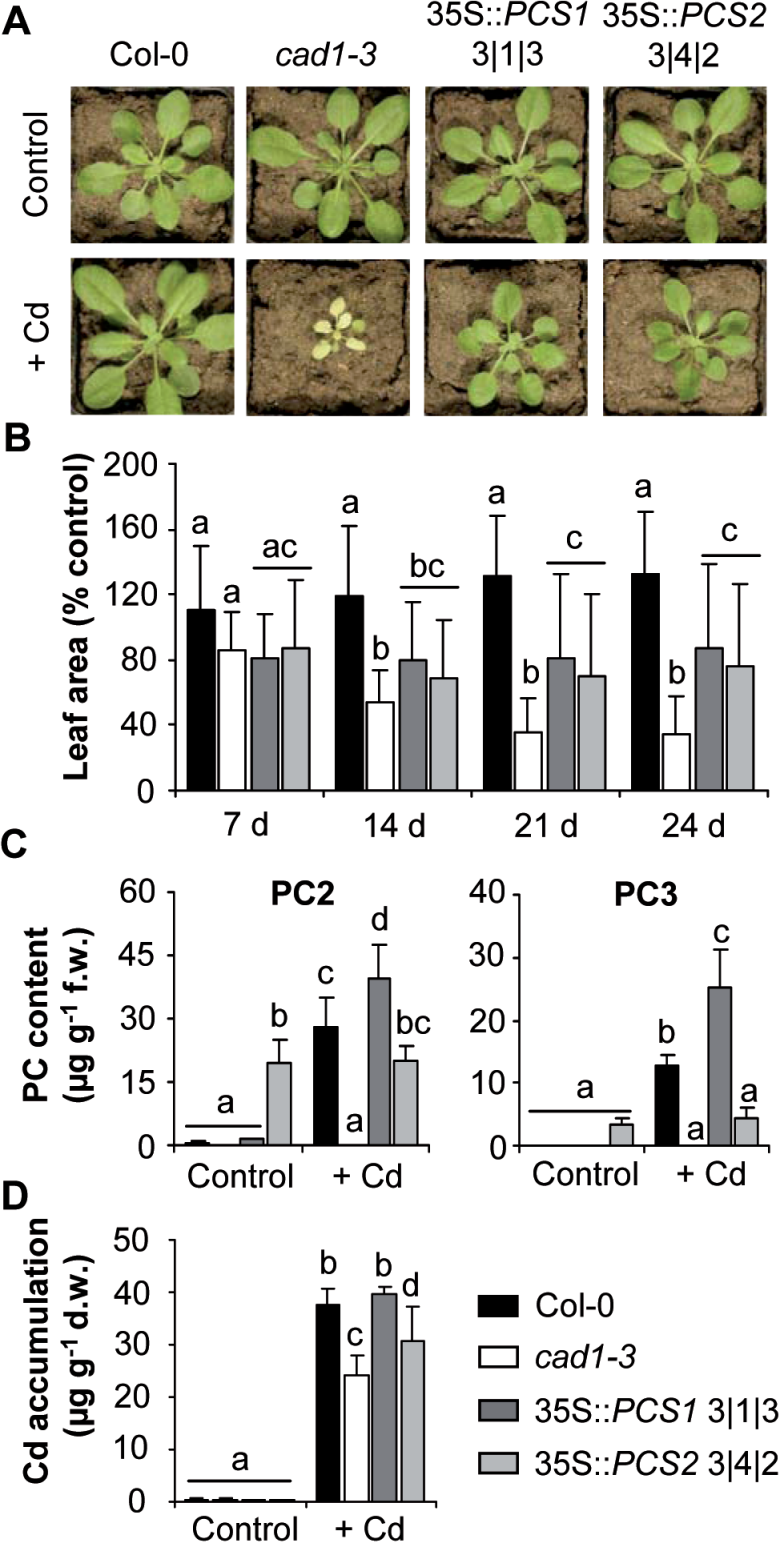
AtPCS2 expression results in constitutive PC accumulation and rescues growth of *cad1-3* on Cd-contaminated soil. Ten-day-old *A. thaliana* seedlings of wild-type Col-0, the *AtPCS1* mutant *cad1-3*, and lines overexpressing AtPCS1 or AtPCS2 in the *cad1-3* background were transferred to control soil or to artificially Cd-contaminated soil. (A) Pictures of plants after 24 d of growth on control soil (top) or soil spiked with 7.5mg Cd^2+^ kg^–1^ (bottom). (B) Leaf area was quantified weekly during the course of the experiment. (C, D) Leaf material was pooled and assayed for PC accumulation via UPLC-ESI-QTOF-MS (C) and Cd accumulation using inductively coupled plasma optical emission spectroscopy (D). Data represent means±SD of two independent experiments (*n*=4). Statistical analyses were performed via two-way ANOVA and data were grouped with Tukey’s 95% confidence interval. Percentage values were transformed prior to analysis.

Wild-type Col-0, the *cad1-3* mutant and the transgenic lines with stronger expression, 35S::*AtPCS1* 3|1|3 and 35S::*AtPCS2* 3|4|2, were cultivated on control mineral soil and soil spiked with Cd. After 24 d of growth on this contaminated soil, the Cd hypersensitivity of the *cad1-3* mutant was obvious from the strong growth reduction and the leaf chlorosis (Fig. 6A). Plant growth was quantified based on the monitoring of leaf area throughout the course of the experiment (Fig. 6B). In order to account for the variation in the plant size between the two independent experiments, leaf area of plants grown on Cd-contaminated soil was expressed relative to the growth of control plants on soil without additional heavy metals. In this way, a growth reduction of up to 65% was determined for *cad1-3*, while Col-0 showed an even better growth on the Cd-contaminated soil than on the control soil. Remarkably, overexpression of *AtPCS1* and *AtPCS2* had an almost equally strong positive effect on growth of *cad1-3* plants on Cd soil. Plants were clearly less chlorotic, and growth was impaired by only 15 and 25%, respectively, after d 24. Differences between the two transgenic lines were not significant, whereas already at d 21 the differences between wild-type, *cad1-3* mutant, and the overexpression lines were highly significant with *P*<0.001 for all comparisons. Thus, complementation by *AtPCS1* and *AtPCS2* was not complete.

PCS activity is well known to influence Cd accumulation in plant tissues (Howden and Cobbett, 1992; Howden *et al.*, 1995*a*; Pomponi *et al.*, 2006; Li *et al.*, 2007; Tennstedt *et al.*, 2009). Cd concentrations in leaf material of the soil-grown plants were determined using inductively coupled plasma optical emission spectroscopy (Fig. 6D). In plants cultivated on control soil, no Cd was detected. On Cd-contaminated soil, *cad1-3* accumulated about 35% less Cd than Col-0. Both *AtPCS1* and *AtPCS2* overexpression resulted in an increase in leaf Cd levels. Full reversion to wild-type levels, however, was found only in the 35S::*AtPCS1* line.

### AtPCS2 shows higher activity than AtPCS1 in the absence of the activating metal Cd

When PC accumulation was analysed, PC2 was again detected even in wild-type plants not exposed to metal stress (Fig. 6C). In contrast, neither PC2 nor PC3 above the LLOD was found in *cad1-3* leaves, regardless of soil conditions. 35S::*AtPCS1* 3|1|3 exhibited higher PC2 and PC3 levels than Col-0 after growth on Cd-contaminated soil, even though it did not show full complementation of the Cd-hypersensitive phenotype of *cad1-3.* Plants overexpressing *AtPCS2* accumulated nearly as much PC2 as Col-0 when grown on Cd-contaminated soil. In contrast, PC3 levels were significantly lower. Interestingly, among plants on control soil, the 35S::*AtPCS2* line showed the strongest accumulation of PC2 and PC3. In fact, concentrations were equally high under both conditions. This was very different from Col-0 and 35S::*AtPCS1* 3|1|3, where growth in the presence of Cd caused 30-fold or 40-fold increases in PC2 production, respectively.

**Fig. 7.**
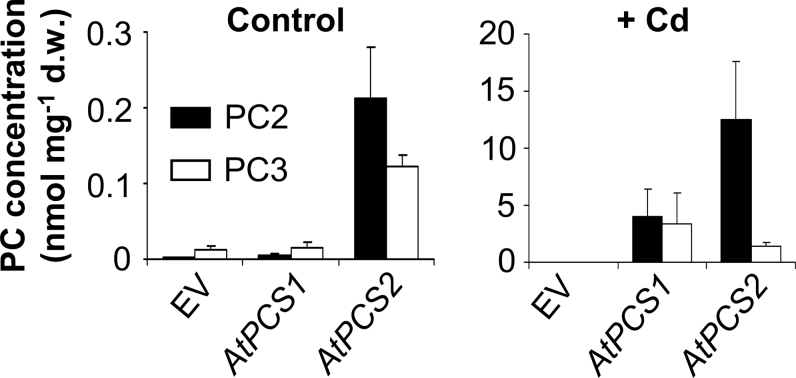
PC accumulation in *Schizosaccharomyces pombe* Δ*pcs* cells expressing AtPCS1 or AtPCS2. *Schizosaccharomyces pombe* cells carrying a construct with *AtPCS1*, *AtPCS2*, or the empty vector (EV) were grown overnight in EMM containing 20 µM thiamine, i.e. under conditions suppressing *PCS* expression. The cells were washed and inoculated to an OD_600_ of 0.4 in EMM without thiamine to induce *PCS* expression in the presence or absence of 10 µM CdCl_2_. After 6h of cultivation, the cells were harvested and PCs were extracted, labelled with mBrB, and quantified using UPLC-ESI-QTOF-MS. Data represent means±SD of two independent experiments (*n*=8).

Elevated PC production in *AtPCS2*-overexpression lines in the absence of additional Cd has been detected previously (Supplementary Fig. S4). These observations suggested that AtPCS2 activity is less responsive to Cd than AtPCS1 activity. In order to test this more directly, i.e. in the absence of plant factors potentially influencing enzyme activity, an attempt to characterize recombinant purified AtPCS2 was made. However, the protein could never be obtained in an active form. Therefore, heterologous expression of AtPCS1 and AtPCS2 in *Schizosaccharomyces pombe* Δ*pcs* mutant cells was compared instead (Supplementary Fig. S5 at *JXB* online). Again, AtPCS2-dependent accumulation of PC2 and PC3 in the absence of Cd^2+^ was observed that was stronger than the respective AtPCS1-dependent accumulation (Fig. 7). Levels of PC2 and PC3 were 50-fold and 8-fold higher, respectively, in AtPCS2-expressing cells than in AtPCS1-expressing cells under control conditions. Furthermore, a lower efficiency of AtPCS2 with respect to catalysing PC3 synthesis was also confirmed. The PC2:PC3 ratio in Cd^2+^-treated cells was about 1.1 for AtPCS1 and about 8.7 for AtPCS2. Because constitutive AtPCS2-dependent PC2 formation was observed in the liquid seedling assay with Fe being the only micronutrient present, possible activation of AtPCS2 by Fe was tested. However, no increase in PC levels in cells treated with an excess of Fe (20 and 100 µM) was detected. PC2 concentrations remained at around 0.2 nmol mg^–1^ of dry weight.

## Discussion

The synthesis of PCs is well established as the major detoxification mechanism for Cd, As, and Hg (Cobbett and Goldsbrough, 2002). Given this function, however, it has been mysterious as to why *PCS* genes are so widespread among higher plants and expressed constitutively even in organs such as leaves, which are rarely if at all exposed to toxic non-essential metals (Rea *et al.*, 2004). In fact, complex formation with PCs and reduced glutathione in the roots has been shown to efficiently restrict movement of toxic metals to the shoot (Chen *et al.*, 2006; Li *et al.*, 2006).

The question can be extended to the existence of at least two *PCS* genes, which appears to be common in the genomes of higher plants (Fig. 1). Available evidence suggests two additional functions of PCS enzymes that could explain their wide occurrence and constitutive expression (Clemens and Peršoh, 2009; Rea, 2012). First, PC synthesis has been implicated in the homeostasis of the essential micronutrient Zn (Tennstedt *et al.*, 2009; Adams *et al.*, 2011). Secondly, AtPCS1 was shown to catalyse the deglycylation of glutathione *S*-conjugates (Grzam *et al.*, 2006; Blum *et al*., 2007, 2010). This second activity is possibly underlying the role of AtPCS1 in mounting innate immune responses in *A. thaliana* seedlings (Clay *et al.*, 2009).

With the aim of further elucidating the physiological functions of PCSs, the activities of the protein encoded by the second *PCS* gene in *A. thaliana*, *AtPCS2*, were analysed. The data presented in this study showed that it contributes to PC synthesis in *A. thaliana*, especially in the absence of metal excess, is able to rescue the Cd hypersensitivity of mutants lacking functional AtPCS1, and appears to possess enzymatic properties distinct from AtPCS1.

The mutant *cad1-3* was initially characterized as devoid of PC accumulation (Howden *et al.*, 1995*b*). This was later confirmed (Cazalé and Clemens, 2001), prompting the question as to the physiological role of the second *PCS* gene in *A. thaliana*, *AtPCS2*, which was found to encode a functional PCS when expressed in yeast. Therefore, a possible AtPCS2-dependent PC synthesis in *cad1-3* was re-evaluated employing a more sensitive PC detection method than previously available. In recent years, the use of TCEP as a reductant (Rijstenbil and Wijnholds, 1996; Thangavel *et al.*, 2007; Minocha *et al.*, 2008) and of UPLC separation, coupled to ESI-QTOF-MS, as a method for PC detection have been reported (Bräutigam *et al.*, 2010). Assessment of the UPLC-ESI-QTOF-MS platform employed and possible matrix effects (Fig. 2) yielded LLODs for PC2 and PC3 that were about three orders of magnitude lower than those initially reported for HPLC-based analysis of *A. thaliana* extracts (Howden *et al.*, 1995*b*). When such values were reported in more recent studies, they were between approximately 2-fold (Minocha *et al.*, 2008) and 20-fold (Simmons *et al.*, 2009) higher. In order to achieve maximum sensitivity, the mass range was restricted to common values of *m*/*z* 300–2000 and the analysis was focused on PC2 and PC3, which are by far the most abundant PCs.

Thiol analysis of derivatized extracts of plants cultivated hydroponically and supplied with all required micronutrients, but in the absence of any potentially toxic metal excess, confirmed constitutive PC synthesis in roots and to a much lesser extent in leaves, which is in accordance with the physiological roles of PC synthesis beyond metal detoxification (Rea, 2012). Furthermore, these experiments yielded unequivocal detection of PC2 in the roots of *cad1-3* (Fig. 3A). This could be due either to residual AtPCS1 activity in *cad1-3* or to AtPCS2 activity. The latter interpretation is consistent with the *cad1-3* mutation affecting an amino acid that is 100% conserved in PCS proteins across kingdoms, as well as the absence of the *AtPCS1* transcript in *cad1-3* (Ha *et al.*, 1999). Furthermore, it is supported by two observations. First, an *atpcs2* exon insertion line showed lower PC2 levels than the parental ecotype Ws. Secondly, and most importantly, no PC2 was detectable in the roots of the *cad1-3 atpcs2* double mutant that was generated. These observations were confirmed for roots of Cd^2+^-treated plants, which as expected showed strong increases in PC accumulation dependent on the Cd^2+^ dose. In contrast, no evidence was found for AtPCS2-dependent PC3 synthesis or leaf PC2 synthesis in *cad1-3*. The latter is consistent with the finding that an AtPCS2–GFP fusion protein expressed under the control of the endogenous *AtPCS2* promoter yielded signals only in the root tip (Blum *et al.*, 2010). The surprising detection of trace amounts of PC2 in the leaves of the *cad1-3 atpcs2* double mutant upon Cd^2+^ treatment might indicate the existence of another as-yet-unknown enzymatic activity that results in PC formation. This would also explain the similar trace amounts of PC2 in the leaves of Cd-treated *cad1-3* plants (Fig. 3B).

The evidence for AtPCS2-dependent PC synthesis *in vivo* prompted testing for a possible complementation of the *cad1-3* mutant phenotype by *AtPCS2* overexpression. Hence, transgenic lines with different levels of AtPCS2 protein were generated and compared with lines overexpressing *AtPCS1*. The most commonly used metal tolerance system, cultivation on vertical agar plates, did not yield any indication for rescue by AtPCS2, while expression of AtPCS1 fully complemented *cad1-3* (Supplementary Fig. S3). In contrast, partial rescue at least in the more strongly expressing AtPCS2 line was indicated when Cd tolerance was assayed in an alternative system: growth of seedlings in liquid medium (Fig. 5). As a result of this finding, a tolerance assay that was far closer to natural conditions was established, i.e. growth in mineral soil spiked with Cd. The concentration of 7.5mg kg^–1^ is only three times higher than the upper limit of the range of background levels in topsoils (EFSA, 2009) and is within the reported span for agricultural soils in some countries (McLaughlin *et al.*, 1999; Pan *et al.*, 2010). Col-0 plants were not affected by this Cd level in the soil. In fact, leaf area as an indicator for growth was even slightly elevated when compared with plants cultivated on control mineral soil (Fig. 6A, B). In contrast, *cad1-3* plants developed chlorotic leaves and growth was severely impaired. This difference between Col-0 and *cad1-3* demonstrated the essential role of PC synthesis for tolerating levels of Cd contamination that can naturally be encountered by plants outside metal-rich habitats. Furthermore, the lower Cd accumulation in *cad1-3* leaves relative to Col-0 (Fig. 6D) provided evidence for the contribution of PC synthesis to Cd accumulation under such conditions. This is highly relevant in light of the widespread background contamination of agricultural soils with Cd and the health risks associated with human intake of Cd through plant-derived food (Nawrot *et al.*, 2010; Clemens *et al.*, 2013; Uraguchi and Fujiwara, 2013).

Interestingly, *AtPCS1* and *AtPCS2* expression rescued *cad1-3* growth on Cd soil to the same extent. After 21 and 24 d of growth, the leaf areas of the two tested transgenic lines were significantly higher than the leaf areas of *cad1-3*. Lack of full complementation, i.e. slower growth than wild type on Cd soil, is in line with earlier observations suggesting that PCS overexpression does not result in a gain of Cd tolerance and in some cases there is even in a reduction (Lee *et al*., 2003*a*,*b*; Wojas *et al.*, 2008). The latter was found not only on Cd soil but also in the liquid seedling assay (Fig. 5).

Contrasting PC accumulation patterns of plants overexpressing either AtPCS1 or AtPCS2 suggested at least two distinct differences in enzymatic properties of the proteins. AtPCS2 appeared to be less efficient in synthesizing PC3, possibly due to a reduced efficiency in accepting PC2 as a substrate in place of reduced glutathione. Moreover, AtPCS2-expressing lines accumulated higher levels of PC2 in the absence of any metal excess both in the liquid seedling assay and on soil (Fig. 6C and Supplementary Fig. S4). This does not correspond with what is considered a hallmark of PCSs, namely activation of the constitutively expressed proteins by metal excess (Grill *et al.*, 1989; Vatamaniuk *et al.*, 2000). In fact, 35S::*AtPCS2* plants did not show any increase in PC2 or PC3 levels in leaves of plants grown on Cd soil (Fig. 6C), even though these plants clearly accumulated Cd. Cd^2+^ ions are among the most potent activators of AtPCS1 (Grill *et al.*, 1987; Ha *et al.*, 1999; Vatamaniuk *et al.*, 2000). This could be seen in the strongly elevated PC2 and PC3 levels of 35S::*AtPCS1* plants when grown on Cd-spiked soil. Thus, according to data obtained in different experimental systems, AtPCS2 appeared to show a PCS activity that, in contrast to the well-established knowledge for AtPCS1, was not stimulated by Cd *in planta*. Lack of AtPCS2 activation in *Schizosaccharomyces pombe* cells exposed to an excess of Fe, the only micronutrient present in the liquid seedling assay, argues against activation by other metals.

Attempts to further elucidate these differences in enzyme properties with recombinant purified proteins were not successful. While purification of active AtPCS1 was possible as reported by other groups (Vatamaniuk *et al.*, 2000), trials to obtain active soluble AtPCS2 failed even when using the same tags, vectors, cloning strategies, and *Escherichia coli* strains (data not shown). As the C-terminal part of the PCS protein has been shown to influence the enzyme stability (Ruotolo *et al.*, 2004), the insolubility of the AtPCS2 protein might be a consequence of the 90bp deletion in exon 8, which causes a major difference in the AtPCS1 protein (Ha *et al.*, 1999). Thus, AtPCS1 and AtPCS2 activities were instead compared following expression in *Schizosaccharomyces pombe* Δ*pcs* cells. The results again confirmed higher PC2 accumulation in the absence of Cd^2+^ treatment due to stronger constitutive activity of AtPCS2 relative to AtPCS1 (Fig. 7). Using this heterologous expression system, it should be possible to dissect the structure–function relationships responsible for the differences in metal activation between AtPCS1 and AtPCS2.

Taken together, the data presented in this study establish constitutive PCS activity, i.e. catalysis of PC formation in the absence of any potentially toxic metal excess, for AtPCS2 *in planta*. When overexpressed, this activity is sufficient to at least partially rescue the severe Cd hypersensitivity of the *cad1-3* mutant. However, AtPCS2-dependent constitutive PC2 formation in *cad1-3* is low relative to the impact of AtPCS1, which is apparent from the PC2 accumulation in the roots of *atpcs2* mutant plants under control conditions (Fig. 3A). Most likely, the small AtPCS2 contribution is due to spatially restricted weak expression. It is not sufficient to confer Cd tolerance, as is obvious from the *cad1-3* phenotype in the presence of Cd (Figs 5 and 6A). In addition, this phenotype might in part be attributable to the reduced PC3 formation by AtPCS2, because a rising chain length leads to a gain in pH stability of PC–Cd complexes (Satofuka *et al.*, 1999) and accordingly to an increased binding affinity of PCs to metal ions (Loeffler *et al.*, 1989). Furthermore, apart from slightly lower PC2 accumulation, no discernible differences between *atpcs2* and Ws-0 were detected. This and the constitutive activity suggest a physiological role of AtPCS2 unrelated to metal detoxification. In accordance with recently demonstrated AtPCS1 functions (Clay *et al.*, 2009), future experiments will have to test AtPCS2-dependent effects on glutathione *S*-conjugate metabolism or plant defence.

## Supplementary data

Supplementary data are available at *JXB* online.

Supplementary Fig. S1. Isolation of the homozygous T-DNA insertion line *atpcs2* (FLAG_146G12).

Supplementary Fig. S2. Genotype of *A. thaliana* wild-type lines, *AtPCS1* and *AtPCS2* mutants, and the *PCS* double mutant at the *HMA3* locus.

Supplementary Fig. S3. Rescue of *cad1-3* Cd hypersensitivity in vertical agar plate assays by *AtPCS1* but not *AtPCS2* expression.

Supplementary Fig. S4. PC accumulation in seedlings of *AtPCS1*- and *AtPCS2*-overexpressing lines after 11 d of growth in the presence or absence of Cd^2+^.

Supplementary Fig. S5. Western blot analysis for the detection of AtPCS1 and AtPCS2 expression in *Schizosaccharomyces pombe*.

Supplementary Table S1. Metal contents in the mineral soil type used as control soil for growth experiments.

Supplementary Table S2. Primer sequences used for quantitative real-time PCR.

Supplementary Table S3. Evaluation of the liquid seedling assay.

Supplementary Data

## Abbreviations:

ANOVA: analysis of variance
CAPS: cleaved amplified polymorphic sequence
DTPA: diethylenetriaminepentaacetic acid
EMM: Edinburgh’s minimal medium
EPPS: 4-(2-hydroxyethyl)-piperazine-1-propanesulfonic acid
HA: haemagglutinin
LLOD: lower limit of detection
LLOQ: lower limit of quantification
mBrB: monobromobimane
MES: 2-(*N*-morpholino)-ethanesulfonic acid
NAC: *N*-acetylcysteine
OD: optical density
PC: phytochelatin
PCS: phytochelatin synthase
SD: standard deviation
TCEP: Tris-(2-carboxyethyl)-phosphine
UPLC-ESI-QTOF-MS: ultraperformance electrospray ionization quadrupole time-of-flight mass spectrometry.
