# Supplementary material for: Arabidopsis thaliana phytochelatin synthase 2 is constitutively active in vivo and can rescue the growth defect of the PCS1-deficient cad1-3 mutant on Cd-contaminated soil
Source: J Exp Bot. 2014 May 12;65(15):4241–53. doi: 10.1093/jxb/eru195 (PMC4112630; doi:10.1093/jxb/eru195)
Supplement: Supplementary Data [file supp_65_15_4241__index.html]

 Arabidopsis thaliana phytochelatin synthase 2 is constitutively active in vivo and can rescue the growth defect of the PCS1-deficient cad1-3 mutant on Cd-contaminated soil — Arabidopsis thaliana phytochelatin synthase 2 is constitutively active in vivo and can rescue the growth defect of the PCS1-deficient cad1-3 mutant on Cd-contaminated soil — Supplementary Data 

# *Arabidopsis thaliana* phytochelatin synthase 2 is constitutively active *in vivo* and can rescue the growth defect of the *PCS1*-deficient *cad1-3* mutant on Cd-contaminated soil

## Supplementary Data

Data files

**Files in this Data Supplement:**

- Supplementary Data - Supplementary Data
