# Supplementary material for: Arabidopsis thaliana phytochelatin synthase 2 is constitutively active in vivo and can rescue the growth defect of the PCS1-deficient cad1-3 mutant on Cd-contaminated soil
Source: J Exp Bot. 2014 May 12;65(15):4241–53. doi: 10.1093/jxb/eru195 (PMC4112630; doi:10.1093/jxb/eru195)
Supplement: Supplementary Data [file supp_eru195_jexbot117713_file001.pdf]

Journal of Experimental Botany

Arabidopsis thaliana phytochelatin synthase 2 is constitutively active *in vivo* and can rescue the growth defect of the *AtPCS1*-deficient *cad1-3* mutant on Cd-contaminated soil

Tanja Kühnlenz, Holger Schmidt, Shimpei Uraguchi, Stephan Clemens

Supplementary Figure S1

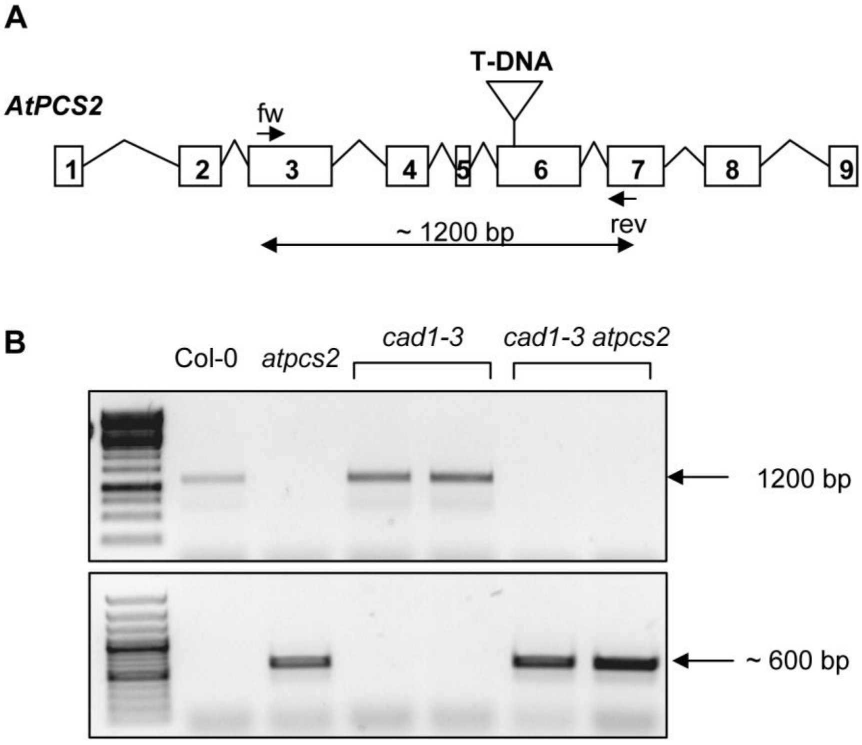

Fig. S1: Isolation of the homozygous T-DNA insertion line *atpcs2*. (A) Gene structure of *AtPCS2* (At1g03980). Numbers in boxes represent exons, lines represent introns. The triangle shows the position of the T-DNA insertion in the line *atpcs2* (FLAG_146G12). Arrows indicate primer binding sites of gene specific primers for detection of the wild type fragment. (B) Detection of the wild type fragment (top) in Col-0 and *cad1-3* and the T-DNA specific fragment (bottom) in *atpcs2* and the *PCS* double mutant *cad1-3 atpcs2*. For primer sequences see "Materials and Methods".

Supplementary Figure S2

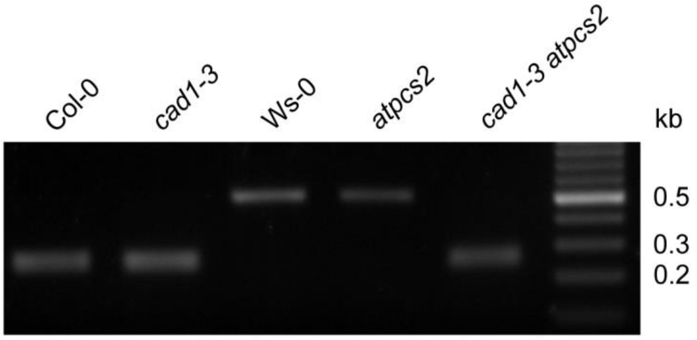

Fig. S2: Genotype of *A. thaliana* wild type lines, *cad1-3* and *atpcs2* mutants and the *PCS* double mutant *cad1-3 atpcs2* at the *HMA3* locus. Leaf material of the *A. thaliana* wild types Col-0 and Ws-0, the *AtPCS1* and *AtPCS2* mutants *cad1-3* and *atpcs2* in the respective wild type background and the *PCS* double mutant *cad1-3 atpcs2*, that was derived from crossing the single mutants, was tested for the genotype at the *HMA3* locus. The single nucleotide deletion present in Col-0 resulting in the generation of a *Dde*I restriction site was visualized by a CAPS marker analysis. For primer sequences see “Materials and Methods”.

Supplementary Figure S3

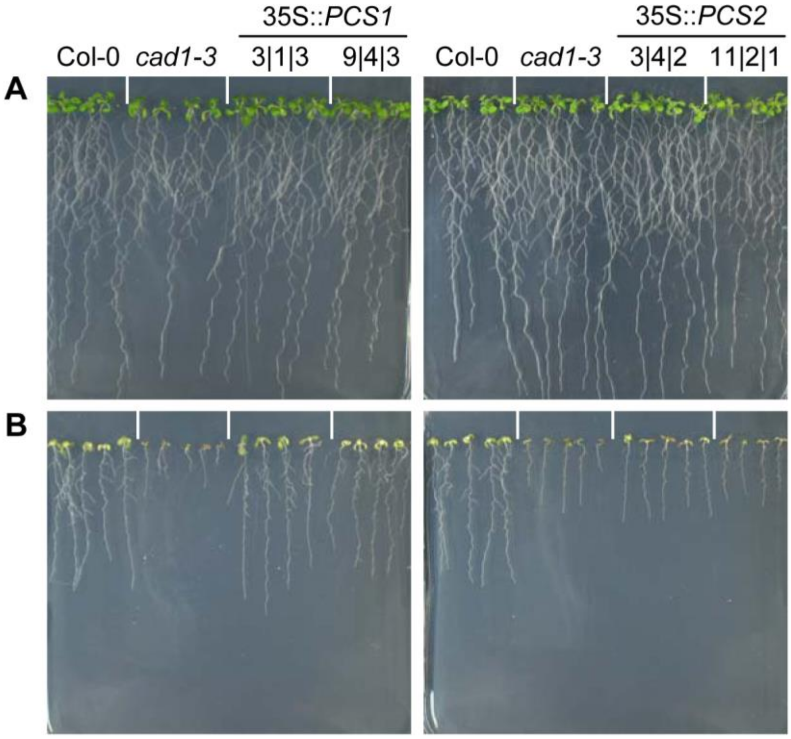

Fig. S3: Rescue of *cad1-3* Cd-hypersensitivity in vertical plate assays by AtPCS1-expression but not by AtPCS2-expression. Seedlings of wild type Col-0, the *AtPCS1* mutant *cad1-3* and lines overexpressing *AtPCS1* or *AtPCS2* in the *cad1-3* background under control of the 35S-promoter were germinated and grown in one-tenth-strength Hoagland medium on agar plates without additional heavy metals (A) or in the presence of 2 µM $CdCl_2$ (B). Representative pictures after 14 d of growth are shown.

Supplementary Figure S4

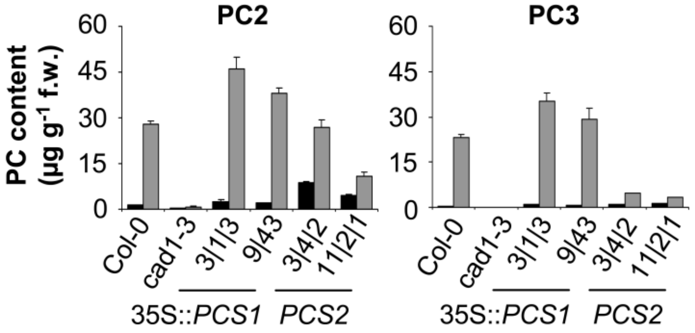

Fig. S4: PC accumulation in seedlings of AtPCS1- and AtPCS2-overexpressing lines. Seedlings of wild type Col-0, the *AtPCS1* mutant *cad1-3* and lines overexpressing *AtPCS1* or *AtPCS2* in *cad1-3* background were germinated and grown in a liquid seedling assay in one-tenth-strength Hoagland medium either without metal addition (black bars) or in the presence of 0.5 µM $CdCl_2$ (grey bars) for 11 days. PC accumulation was assayed by UPLC-ESI-QTOF-MS. Measurements were performed in duplicates. Shown are means ± SD (n=2) of one representative cultivation.

Supplementary Figure S5

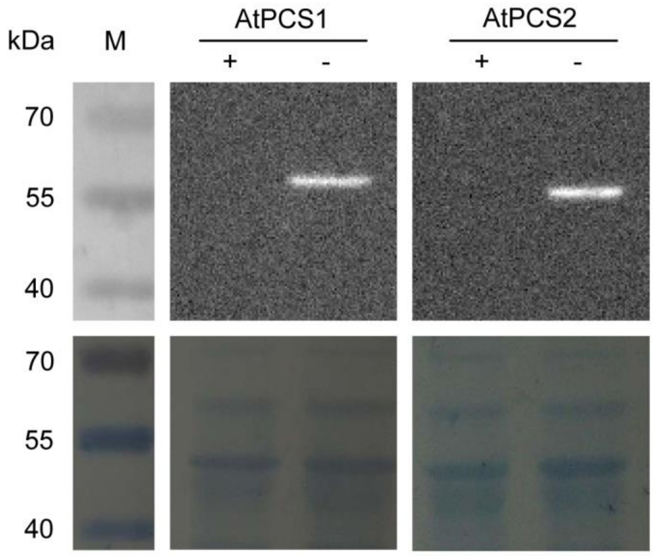

Fig. S5: Detection of AtPCS1 and AtPCS2 protein expression in *S. pombe.* *S. pombe Δpcs* cells carrying the vectors pSGP72-*AtPCS1* or pSGP72-*AtPCS2* were grown in the presence or absence of 20 µM thiamine, i.e. under conditions suppressing or inducing expression, respectively. Protein extracts were analyzed via SDS-PAGE, western blotting and immunostaining. Tagged version of AtPCS1 (58 kDa) and AtPCS2 (55 kDa) were detected in the respective strains in the absence of thiamine using an anti-HA antibody. The amidoblack stained membrane is shown as loading control below the blot.

Supplementary Table S1

Tab. S1: Metal contents in the mineral soil type used as control soil for growth experiments. Extractable and exchangeable metals were analyzed after extraction of the soil with HCl, DTPA or $CaCl_2$ via ICP-OES. (n.q. not quantifiable; n.d. not detectable; * LLOD = 0.01 mg kg^{-1} soil; ** LLOQ = 0.1 mg kg^{-1} soil)

Extraction method	Soil metal content (mg kg^{-1} d.w.)						
	Zn	Fe	Cu	Mn	Ni	Co	Cd
HCl	5.8 ± 0.5	155 ± 11	0.8 ± 0.1	134 ± 17	0.6 ± 0.03	0.8 ± 0.1	n.d.*
DTPA	2.4 ± 0.1	84 ± 9	0.7 ± 0.1	44 ± 7	n.q.**	n.q.**	n.d.*
$CaCl_2$	n.d.*	0.5 ± 0.1	n.d.*	11 ± 2	n.d.*	n.d.*	n.d.*

Supplementary Table S2

Tab. S2: Primer sequences used for quantitative realtime PCR. Primers were adopted from Talke *et al.*, 2006; Bernal *et al.*, 2012 and Deinlein *et al.*, 2012.

**Name**	**Sequence 5'-3'**
ZIP9_fw	ATCACCGTCATAGCCTCTCCATG
ZIP9_rev	CATGACCGGAAACACCACTTCTC
CCH_fw	GTTGGTATGTCATGCCAAGGCT
CCH_fw	CAAATGACTCAACCCCTTCCAT
COX5b-1_fw	GCCAATCGATCAGCCATTTC
COX5b-1_rev	ACCTTCTTCGTCGCAGGAGTCT
EF1α_fw	TGAGCACGCTCTTCTTGCTTTCA
EF1α_rev	GGTGGTGGCATCCATCTTGTTACA

Supplementary Table S3

Tab. S3: Evaluation of the liquid seedling assay. Transcript levels of established molecular markers for micronutrient deficiency were determined by quantitative realtime PCR. Col-0 and *cad1-3* seedlings were cultivated in one-tenth-strength Hoagland medium with all micronutrients (+ME) and in Hoagland medium without micronutrients except for Fe (-ME). Shown are Relative Transcript Levels (RTL) +/- SD of two independent experiments.

		**Mean RTL +/- SD**		
		ZIP9	*COX5b-1*	*CCH*
+ ME	Col-0	5.94 +/- 4.12	84.84 +/- 19.95	55.88 +/- 17.16
	cad1-3	8.40 +/- 4.08	95.54 +/- 15.66	92.19 +/- 20.40
- ME	Col-0	9.61 +/- 6.94	75.58 +/- 12.04	135.28 +/- 36.36
	cad1-3	21.82 +/- 18.29	73.60 +/- 9.33	116.47 +/- 46.62
